# The Saudi Population's Knowledge and Attitude Towards Human Papillomavirus (HPV) Infection and Its Vaccination

**DOI:** 10.7759/cureus.58427

**Published:** 2024-04-16

**Authors:** Salim A Algaadi, Hamad J Aldhafiri, Razan S Alsubhi, Mohammed Almakrami, Nour H Aljamaan, Yazeed A Almulhim

**Affiliations:** 1 Department of Dermatology, College of Medicine, Majmaah University, Al-Majmaah, SAU; 2 Medicine, Majmaah University, Majmaah, SAU; 3 Medicine, College of Medicine, University of Hai’l, Hai’l, SAU; 4 Medicine, University of Szeged Albert Szent-Györgyi Medical School, Szeged, HUN; 5 Medicine and Surgery, College of Medicine, King Faisal University, Alhasa, SAU; 6 Medicine and Surgery, Alzulfi General Hospital, Second Health Cluster, Riyadh, SAU

**Keywords:** chealth education programs, vaccine acceptance, cervical cancer, hpv vaccination, human papillomavirus (hpv)

## Abstract

Background: Human papillomavirus (HPV) infection is a major worldwide public health concern that can result in a range of clinical disorders, including cervical cancer. Saudi Arabia, similar to numerous other nations, has difficulties in facing HPV and its impact on society. The high incidence of cervical cancer in Saudi Arabia continues to be a cause for worry, highlighting the need for the adoption of efficient immunization programs. Nevertheless, public hesitation and inadequate knowledge can hinder the acceptance of vaccines. Evaluating public knowledge and attitudes concerning HPV and its vaccination is essential in order to create focused programs that enhance awareness and increase vaccine acceptance.

Methods: This study was cross-sectional in nature, using data from a sample of 516 Saudi participants 18 years and above. The participants completed online questionnaires that were distributed using Google Forms across social media platforms and ensured anonymity.

Results: A total of 516 participants made up the sample for this study, which had a predominance of females (83.5%, n=431); the majority aged 18-25 years (78.3%, n=404) and most of them (28.3%, n=146) were from the central region. The study results revealed that 43.7% (n=225) of the participants had a good knowledge level while 56.3% (n=291) of them had a poor level of knowledge about the HPV vaccine. A substantial proportion (35.9%, n=185) of the participants had good knowledge that HPV can be transmitted sexually from one person to another with only 30.2% (n=156) of them being aware that HPV is a common infection that causes cervical cancer (41.5%, n=214) and most of them (76.2%, n=393) knew that cervical cancer can be cured particularly when detected in early stages. The results established statistically significant associations between gender, education level, and occupation with p-values <0.005 (0.023, 0.003 and 0.001 respectively) and level of knowledge about the HPV vaccine.

Conclusion: The study emphasizes the necessity of implementing focused health education and vaccination initiatives in Saudi Arabia to enhance understanding and attitudes regarding HPV infection and its vaccine. The results can provide guidance to healthcare professionals, legislators, and public health authorities in creating programs that increase knowledge and acceptance of the HPV vaccine, ultimately decreasing the prevalence of HPV-related diseases in the nation.

## Introduction

Human papillomavirus (HPV) is a major global public health issue, as it is the most prevalent sexually transmitted infection (STI) that can lead to a range of clinical conditions, from harmless warts to cancerous tumors such as cervical, anal, and oropharyngeal malignancies [1,2]. The introduction of preventive vaccinations that specifically target high-risk strains of HPV has the potential to greatly decrease the occurrence of illnesses linked with HPV [3].

Saudi Arabia, amongst other developing countries, is dealing with difficulties in addressing HPV and its consequences. The prevalence of cervical cancer in Saudi Arabia continues to be a cause for worry, necessitating the implementation of efficient immunization programs [4]. The uptake of the HPV vaccine is significantly influenced by parental decisions, particularly for girls aged 11 to 12 years, who are the recommended target group. Catch-up vaccines are also available for older adolescents [5].

Although these preventive strategies are accessible, vaccine adoption can be impeded by public hesitation and gaps in understanding. The acceptability of HPV vaccination in Saudi Arabia is hindered by cultural sensitivities and a lack of information, which act as hurdles [6]. Therefore, it is essential to comprehend the public knowledge and attitudes regarding HPV and its vaccination in order to customize health education and vaccination campaigns that can effectively tackle these obstacles and encourage vaccine uptake [7]. The objective of this study is to examine the knowledge and attitudes of the population in Saudi Arabia regarding HPV infection and its vaccine. The study intends to evaluate the extent of awareness and identify any misconceptions or obstacles that may hinder the acceptance of the vaccine. The results will offer significant knowledge for healthcare practitioners, politicians, and public health authorities to create focused initiatives that can enhance awareness and adoption of the HPV vaccine, thereby lessening the impact of HPV-related illnesses in the kingdom [8].

## Materials and methods

The study utilized a cross-sectional research design to explore the knowledge and attitudes of Saudi participants regarding HPV infection and associated vaccinations, with the goal of preventing cervical cancer in various regions of Saudi Arabia. The study was carried out between June 2023 and September 2023, with 516 Saudi volunteers who were over the age of 18. Participants were recruited via a random distribution of a closed-ended online questionnaire. The questionnaire, validated prior to distribution to ensure the reliability and consistency of the gathered information, was administered through Google Forms and shared on multiple social media platforms. The survey included a total of 16 questions, which were categorized into three sections: six questions regarding sociodemographic data, six questions assessing knowledge about HPV and its vaccination, and four questions regarding attitudes towards the vaccination. The data analysis was conducted using SPSS version 25 (IBM Corp., Armonk, NY, USA). Descriptive statistics were used to provide a summary of the sociodemographic characteristics and the responses to the knowledge and attitude items. Inferential statistics, including chi-square tests, were utilized to analyze the associations between sociodemographic variables such as age, gender, education level, and knowledge regarding HPV vaccination. The threshold for statistical significance was established at a p-value of less than 0.05. Ethical considerations encompassed voluntary participation, participant anonymity, and getting previous ethical approval from the University Ethics Committee at Majmaah University (MUREC-jun.19/COM-2023/23-10).

## Results

A total of 516 participants completed the questionnaire. Table 1 shows the socio-demographic information of the study subjects. The vast majority (83.5%, n=431) of participants were females with more than half (77.1%, n=398) being Saudi nationals and most of the participants (78.3%, n=404) were aged between 18-25 years. 28.3% (n=146) of the participants were from the central region. A substantial proportion (42.2%, n=285) of the participants had vocational school education while more than half of the participants (69.6%, n=359) were students.

**Table 1:** Socio-demographic information of the participants (N=516) Socio-demographic information presented in frequencies (n) and proportion (%)

Socio-demographic information	Category	Frequency and Proportion n (%)
Gender	Male	85 (16.5%)
	Female	431 (83.5%)
Nationality	Saudi	398 (77.1%)
	Non-Saudi	118(22.9%)
Age	18-25	404 (78.3%)
	25-30	37 (7.2%)
	30-40	42 (8.1%)
	40-50	25 (4.8%)
	More than 50	8 (1.6%)
Place of residence	Central	146 (28.3%)
	Eastern	97 (18.8%)
	Northern	49 (9.5%)
	Southern	97 (18.8%)
	Western	127(24.6%)
Education Level	Uneducated	2 (0.4%)
	Elementary School	3 (0.6%)
	Intermediate	11 (2.1%)
	High School	170 (32.9%)
	Vocational school	218 (42.2%)
	Diploma	20 (3.9%)
	Bachelor’s degree	80 (15.5%)
	Post-graduation education	12 (2.4%)
Occupation	Healthcare professional	15 (2.9%)
	student	359 (69.6%)
	Unemployed	87 (16.9%)
	Other	55 (10.6%)

Table 2 depicts the knowledge about HPV, vaccination and cervical cancer of the participants. The findings demonstrate that 39.9% (n=206) of the participants had the knowledge that both genders can be infected with HPV. The vast majority of the participants (94.0%, n=485) had the knowledge that someone might be infected by HPV but not know. Only 35.9% (n=185) of the respondents were aware that HPV can be transmitted sexually from one person to another. Only 30.2% (n=156) of the participants were aware that HPV is a common infection while 41.5% (n=214) of them had knowledge that HPV infection causes cervical cancer. The majority of the participants (76.2%, n=393) were aware that cervical cancer can be cured particularly when detected in early stages.

**Table 2:** Knowledge about HPV, vaccination and cervical cancer Knowledge about HPV, vaccination and cervical cancer presented in frequencies (n) and proportion (%)

Questions	Categories	Frequency and Proportion n (%)
Who can get infected with HPV	Both	206 (39.9%)
	Females	207 (40.1%)
	Males	6 (1.2%)
	I don’t know	97 (18.8%)
Do you know that someone might be infected by HPV, but he/she does not know	Yes	485 (94.0%)
	No	31 (6.0%)
Do you know that HPV can be transmitted sexually from one person to another	Yes	185 (35.9%)
	No	331(64.1%)
Do you know that HPV infection is common	Yes	156 (30.2%)
	No	360 (69.8%)
Do you know that HPV infection can cause cervical cancer	Yes	214 (41.5%)
	No	302 (58.5%)
Do you know that cervical cancer can be cured, specifically when it is detected in the early stages	Yes	393 (76.2%)
	No	123 (23.8%)

**Figure 1:**
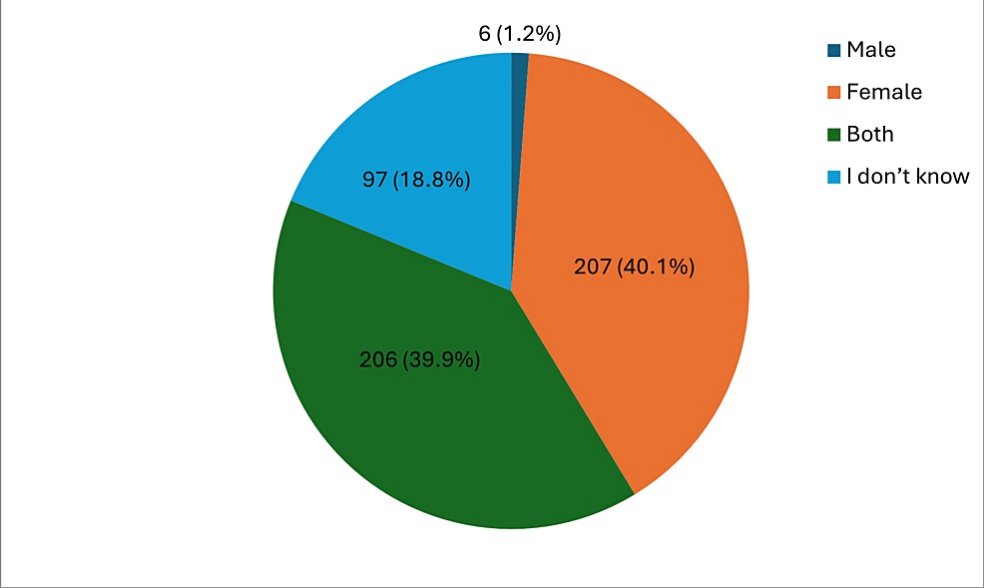
Proportion of participants and their knowledge about HPV infection across gender

Figure 1 illustrates the proportion of participants and their knowledge about the gender groupings that can get infected with HPV. According to the findings, a substantial proportion (206, 39.9%) of the participants correctly reported that both genders can be infected with HPV. A considerable majority (207, 40.1%) of the participants cited females; 97 (18.8%) were not sure while only six (1.2%) cited males. 

Table 3 presents information on the general attitude towards vaccination. The vast majority (82.4%, n=425) of the participants agreed that vaccination is effective in preventing diseases. More than half (70.0%, n=361) were worried about the vaccine’s side effects while 75.9% (n=392) of the participants understood that vaccines are administered to prevent very severe diseases.

**Table 3:** General attitude towards vaccination General attitude towards vaccination among participants presented in frequencies (n) and proportion (%)

Statement	Strongly disagree	Disagree	Agree	Strongly agree
Vaccination is effective to prevent disease	34 (6.5%)	57 (11.1%)	321 (62.2%	104 (20.2%)
I am worried about vaccine’s side effects	40 (7.8%)	115 (22.2%)	284 (55.0%)	77 (15.0%)
Vaccines are administered to prevent very severe disease	40 (7.8%)	84 (16.3%)	278 (53.9%)	114 (22.0%)
Vaccines are administered to prevent sexually transmitted diseases	59 (11.4%)	99 (19.2%)	257 (49.8%	101 (19.6%)

Table 4 depicts the relationship between participants’ demographics such as gender, nationality, age, place of residence, educational level, occupation and level of acceptance of the HPV vaccine. The results established a statistically significant association between gender, education level, and occupation with p-values <0.005 (0.023, 0.003 and 0.001 respectively) and the level of knowledge about the HPV vaccine. There were no statistically significant associations between nationality, age, place of residence, and the level of knowledge about the HPV vaccine (p>0.005).

**Table 4:** The association between socio-demographic information and level of knowledge of the HPV vaccine Association between participants’ demographics and level of knowledge about the HPV vaccine * Significant at p<0.05 level.

Variables	Category	Poor	Good	p value
Gender	Male	50 (58.6%)	35 (41.4%)	0.023*
	Female	216 (50.1%)	215 (49.9%)	
Nationality	Saudi	183 (45.9%)	215 (54.1%)	0.149
	Non-Saudi	68 (57.7%)	50 (42.3%)	
Age	18-25	219 (54.2%)	185 (45.8%)	0.306
	25-30	21 (57.6%)	16 (42.4%)	
	30-40	26 (60.8%)	16 (39.2%)	
	40-50	15 (58.2%)	10 (41.8%)	
	More than 50	5 (56.9%)	3 (43.1%)	
Place of residence	Central	87 (57.2%)	62 (42.8%)	0.210
	Eastern	58 (59.4%)	39 (40.6%)	
	Northern	29 (59.7%)	20 (40.3%)	
	Southern	57 (58.8%)	40 (41.2%)	
	Western	75 (59.1%)	52 (40.9%)	
Education level	Uneducated	1 (69.1%)	1 (30.9%)	0.003*
	Elementary school	2 (67.9%)	1 (32.1%)	
	Intermediate	7 (60.3%)	4 (39.7%)	
	High school	102 (59.8%)	68 (40.2%)	
	Vocational school	124 (56.9%)	94 (43.1%)	
	Diploma	11 (52.5%)	9 (47.5%)	
	Bachelor’s degree	40 (50.1%)	40 (49.9%)	
	Post-graduate	5 (48.7%)	7 (51.3%)	
Occupation	Healthcare profession	6 (42.8%)	9 (57.2%)	0.001*
	Students	177 (49.3%)	182 (50.7%)	
	Unemployed	46 (52.7%)	41 (47.3%)	
	Others	33 (59.6%)	22 (40.4%)	

**Figure 2:**
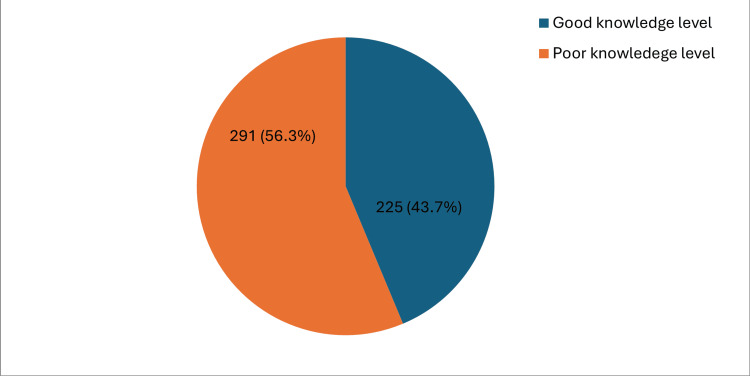
Proportion of good knowledge level and poor knowledge level

Figure 2 illustrates the proportion of participants who had a good knowledge level and a poor knowledge level about HPV vaccines. The study results revealed that 43.7% (n=225) of the participants had a good knowledge level while 56.3% (n=291) of them had a poor level of knowledge about the HPV vaccine.

## Discussion

The study aims to assess the knowledge and attitude towards HPV infection and its vaccination among the Saudi Arabian population. The sample for the current study primarily consisted of participants aged 18-25 years, with a predominance of females; the majority of them were residents of the central region with a vocational education level.

The study results revealed that 43.7% (n=225) of the participants had a good knowledge level while 56.3% (n=291) of them had a poor level of knowledge about the HPV vaccine. The results found a higher proportion of females (215, 49.9%) with good knowledge about HPV vaccines than male participants (35, 41.4%) although it was not statistically significant. The study found a high proportion (185, 45.8%) of good knowledge among participants aged between 18-25 years. In terms of education level, the study revealed a statistically significant association between education and knowledge about HPV infection and its vaccines. Participants with post-graduate and bachelor’s degrees had better knowledge about HPV infections and its vaccines than those with other education levels. Additionally, participants who were healthcare professionals demonstrated significantly higher knowledge of HPV infection and its vaccines than participants in other occupations. A substantial proportion (35.9%, n=185) of the participants were aware that HPV can be transmitted sexually from one person to another with only 30.2% (n=156) of them being aware that HPV is a common infection that causes cervical cancer (41.5%, n=214) and most of them (76.2%, n=393) knew that cervical cancer can be cured particularly when detected in early stages. The current study reveals a considerable knowledge gap with HPV which is congruent with the study done by Al-Shaikh, which revealed that Saudi women showed a high degree of awareness regarding HPV. However, their knowledge regarding the HPV vaccine and its efficacy in preventing cervical cancer was significantly lower [9]. Moreover, a cross-sectional study carried out in the eastern region of Saudi Arabia revealed a connection between sociodemographic variables, such as gender, age, education, employment, and number of children, and the level of awareness and willingness to accept the HPV vaccine. This implies that specifically addressing these factors could greatly enhance both knowledge and vaccination rates [10]. Furthermore, a comparable outcome was obtained in the southwestern region, indicating a lack of understanding among the participants. Although the participants in this study had limited knowledge, around half of the respondents expressed curiosity about the HPV vaccine and displayed a neutral stance towards its safety, effectiveness, and cost [11]. The same results have also been noticed around the world. Roy and Tang revealed low levels of understanding of HPV and vaccination in Kolkata, India [12]. Similarly, studies conducted in several countries have consistently indicated that participants have insufficient information about HPV and vaccination. The most influential factors contributing to this lack of understanding are the participants' level of education and age [12-15]. 

Meanwhile a cross-sectional study conducted in Indonesia showed high knowledge, with most statements (87.5%) that were used in the questionnaire being understood correctly by the majority of respondents. Regarding attitude, most respondents agreed about the importance of the protection effect of HPV vaccination for their children. Regarding decision-making related to vaccines, many respondents knew that the HPV vaccine is safe and effective, and there are no religious limitations to taking vaccines; however, the price of the vaccine and respondents’ spouses were the only factors that affected their decision [16].

In addition to the previously mentioned studies, Alsubaie et al. conducted a cross-sectional study to assess the knowledge and attitudes towards HPV infection and vaccination among Saudi females. The study revealed that there were gaps in knowledge among the participants, with some misconceptions about HPV transmission and prevention. However, the majority of the participants showed positive attitudes towards HPV vaccination, indicating a favorable disposition towards the vaccine [17].

Similar to this, Alrowais et al. studied Saudi women's attitudes and knowledge on HPV and the HPV vaccine. The findings indicated a lack of thorough understanding regarding HPV and its relationship with cervical cancer. The participants did, however, show favorable views toward the vaccine and acknowledged its potential to prevent HPV-related diseases such as cervical cancer [18].

In the current study, the poor level of knowledge regarding HPV and vaccination in the Kingdom of Saudi Arabia can be attributed to insufficient health education from the side of primary health care [19]. As such, there is a need for increased public awareness about HPV vaccines in order to increase the uptake of vaccines among the population.

A significant majority (82.4%, n=425) of the participants had a positive attitude towards vaccination due to their effectiveness in preventing diseases. While a substantial proportion (75.9%, n=392) of the participants understood that vaccines were administered to prevent very severe diseases, 70.0% (n=361) of them were worried about the vaccine’s side effects. The study revealed statistically significant associations between gender, education level, and occupation with p-values <0.005 (0.023, 0.003 and 0.001 respectively) and the level of knowledge about the HPV vaccine.

The significant constraint and limitations in this investigation were the employment of a cross-sectional study design which can only establish the relations between factors but not causalities. As this involved the use of online questionnaires, the data collection may threaten the credibility of the gathered data. Also, considering that the data obtained was self-reported, there was a possibility of measurement bias given that self-reporting depends on participants’ education level and memorizing capabilities.

## Conclusions

Overall, the study revealed a substantial level of knowledge with less than half of the participants having good knowledge about the HPV vaccines. The majority of the participants had a positive attitude towards HPV infection and its vaccines; however, a significant proportion of them were worried about its side effects. Female participants with bachelor's and post-graduate degrees as well as those working in healthcare demonstrated good knowledge about HPV infection and its vaccines. The study revealed a considerable knowledge gap about the HPV causes, transmission and possible complications of HPV vaccination. Therefore, we recommend concerted efforts be made by the medical community to increase the knowledge and acceptance of HPV vaccination among the population.
